# Evaluation of the Mini-Mental State Examination and the Montreal Cognitive Assessment for Predicting Post-stroke Cognitive Impairment During the Acute Phase in Chinese Minor Stroke Patients

**DOI:** 10.3389/fnagi.2020.00236

**Published:** 2020-08-06

**Authors:** Yueli Zhu, Shuai Zhao, Ziqi Fan, Zheyu Li, Fan He, Caixiu Lin, Win Topatana, Yaping Yan, Zhirong Liu, Yanxing Chen, Baorong Zhang

**Affiliations:** ^1^Department of Neurology, The Second Affiliated Hospital, Zhejiang University School of Medicine, Hangzhou, China; ^2^Zhejiang Provincial Center for Disease Control and Prevention, Hangzhou, China; ^3^Department of Neurology, Affiliated Hangzhou First People’s Hospital, Zhejiang University School of Medicine, Hangzhou, China

**Keywords:** mini-mental state examination, montreal cognitive assessment, prediction, cognitive impairment, acute stroke

## Abstract

**Objective**: To assess the value of the Mini-Mental State Examination (MMSE) and the Montreal Cognitive Assessment (MoCA) during acute phase in predicting post-stroke cognitive impairment (PSCI) at 3–6 months.

**Methods**: We prospectively recruited 229 patients who had suffered their first-ever ischemic stroke. PSCI was determined in 104 of these patients by a comprehensive neuropsychological battery performed at 3–6 months. Receiver operating characteristic (ROC) curve analysis was then performed to compare the discriminatory ability of the MMSE and MoCA. Also, we applied a decision tree generated by the classification and regression tree methodology.

**Results**: In total, 66 patients had PSCI when evaluated 3–6 months after the onset of minor stroke. Logistic regression analysis revealed that education, body mass index (BMI), and baseline MoCA scores were independently associated with PSCI. ROC curve analysis showed that the ability to predict PSCI was similar when compared between baseline MoCA scores [area under curve (AUC), 0.821; 95% confidence interval (CI), 0.743–0.898] and baseline MMSE scores (AUC, 0.809; 95% CI, 0.725–0.892, *P* = 0.75). Both MMSE and MoCA exhibited similar predictive values at their optimal cut-off points (MMSE ≤27; sensitivity, 0.682; specificity, 0.816; MoCA ≤21; sensitivity, 0.636; specificity, 0.895). Classification and regression tree-derived analysis yielded an AUC of 0.823 (sensitivity, 0.803; specificity, 0.842).

**Conclusion**: When applied within 2 weeks of stroke, the MMSE and MoCA are both useful and have similar predictive value for PSCI 3–6 months after the onset of minor stroke.

## Introduction

Cognitive impairment is a common sequel after stroke and is known to be associated with poor functional outcomes and even death (Oksala et al., 2009; Gottesman and Hillis, 2010; Jokinen et al., 2015). Post-stroke cognitive impairment (PSCI) is the term used to define any cognitive consequence that occurs after stroke in cases with no evidence of any major cognitive decline before the stroke (Leys et al., 2005; Chander et al., 2017). The prevalence of PSCI ranges from 20% to 80% and is known to show variation according to biogeographical region, race, and diagnostic criteria (Sun et al., 2014). The assessment of cognitive function in the acute phase of stroke can help predict and early identify PSCI patients. Also, timely intervention for these patients may slow the progression of cognitive impairment. The American Heart Association/American Stroke Association recommended that all stroke patients should be screened for cognitive deficits before discharge (Winstein et al., 2016). Therefore, there is an urgent need to develop methods that are capable of identifying patients who are at risk of PSCI, particularly in the acute phase after stroke.

A comprehensive neuropsychological battery is recommended to diagnose PSCI. The Mini-Mental State Examination (MMSE; Folstein et al., 1975) and Montreal Cognitive Assessment (MoCA; Nasreddine et al., 2005), on the other hand, have been used as screen tools for PSCI patients. MMSE is widely used but has been reported to have a low sensitivity for detecting cognitive impairment after stroke (Blake et al., 2002; Nys et al., 2005). In 2006, the National Institute of Neurological Disorders and Stroke–Canadian Stroke Network recommended the use of MoCA to screen PSCI (Hachinski et al., 2006). MoCA is a good predictor of cognitive impairment 6–9 months after stroke (Salvadori et al., 2013). In 2011, a study of 95 patients indicated that both the MMSE and MoCA were moderately sensitive to PSCI (Godefroy et al., 2011). However, this study evaluated patients at a mean post-stroke interval of 24.1 ± 6.4 days, which was too early to identify PSCI. As reported previously, cognitive improvements mostly occur in the first 3 months after stroke (Nijsse et al., 2017). A subsequent study showed that the MMSE and MoCA were both good screening tools for cognitive impairment at 3 months post-stroke (Cumming et al., 2013). MoCA, on the other hand, was reported to be particularly useful to discern PSCI in patients whose cognitive deficits were undetectable with the MMSE (Suda et al., 2020). To compare the prognostic value of the MMSE and MoCA for the detection of cognitive impairment 3–6 months after stroke, a study was conducted to evaluate the MMSE and MoCA in the acute phase of stroke patients (Dong et al., 2012). However, this study aimed to identify patients with moderate-to-severe PSCI; in other words, impairment in at least three cognitive domains. Furthermore, both of these previous studies recruited patients who had experienced stroke previously; this previous insult may already have caused a deterioration in cognitive function. To the best of our knowledge, no previous study has attempted to compare the predictive value of the MMSE and MoCA for cognitive impairment at 3–6 months post-stroke in Chinese patients.

A range of risk factors has been associated with poorer levels of cognitive impairment after stroke, including age, low educational level, hypertension, diabetes mellitus, hyperlipidemia, hyperhomocysteinemia, and smoking (Jacquin et al., 2014; Lu et al., 2016). Identifying the specific risk factors that are associated with PSCI would significantly improve the ability to predict PSCI.

The present study aimed to investigate the prognostic value of the MMSE and MoCA for predicting cognitive impairment 3–6 months after the first stroke and to investigate the risk factors associated with this form of cognitive impairment.

## Materials and Methods

### Patients

All patients referred to our hospital within the acute phase of their first-ever ischemic stroke (within 2 weeks of onset) between February 2018 and March 2019 were considered for inclusion. Patients over 18 years of age in whom acute cerebral infarction was confirmed by magnetic resonance imaging (MRI), with corresponding clinical symptoms, were included. The Informant Questionnaire on Cognitive Decline in the Elderly was also completed by the relatives of each patient to identify symptoms associated with cognitive decline before the onset of stroke. None of the patients recruited had complained to their relatives about cognitive decline, or the potential effect of cognitive decline on daily life activities, before the onset of stroke. Patients were excluded if they had been diagnosed with cognitive decline before their stroke and had an Informant Questionnaire on Cognitive Decline in the Elderly score ≥ 3.44 (Jorm, 2004); had severe visual or communication disturbances that prevented completion of the cognitive assessments; had experienced a previous stroke, traumatic brain injury, neurological, or severe psychiatric diseases that may influence cognition; had been treated with intravenous thrombolysis or mechanical thrombectomy; or if the patient failed to provide informed consent.

The Human Ethics Review Committee of the Second Affiliated Hospital, Zhejiang University School of Medicine, approved this study. Written informed consent was obtained from all patients or their legally responsible representatives.

### Clinical Assessment

For each patient, we collected a range of data on admission relating to demographics (age, sex, and level of education), cardiovascular risk factors [hypertension, diabetes mellitus, hyperlipidemia, atrial fibrillation, smoking history, and body mass index (BMI)], lesion location, the severity of the stroke, and the ApoE genotype. The severity of stroke was assessed using the National Institutes of Health Stroke Scale (NIHSS) and the modified Rankin Scale (mRS). Laboratory test results were obtained, including glycosylated hemoglobin and homocysteine levels.

### Cognitive Assessment

On admission, all recruited patients were asked to complete the Chinese version of the MMSE and the Chinese Changsha version of the MoCA. One point was added to the MOCA score for patients with an education of less than 6 years. During follow-up (3–6 months after stroke), all patients were asked to complete the MMSE and MoCA again; also, we administered a comprehensive neuropsychological battery that evaluated four cognitive domains: (1) language (Boston Naming Test; Mack et al., 1992); (2) visuoconstruction (Clock Drawing Test; Sunderland et al., 1989); (3) verbal memory (Auditory Verbal Learning Test; Mungas, 1983); and (4) executive function/attention (Trail Making Test; Moses, 2004). Impairment was defined by the attainment of a result that was 1.5 standard deviations below the standardized mean. The diagnosis of PSCI required deficits in at least one domain, as assessed by the neuropsychological battery.

The MMSE and MoCA were carried out at baseline by a medical student, following an appropriate training period. A follow-up assessment was performed by another trained medical student who was blinded to the baseline cognitive assessment.

### Statistical Analysis

Patients were classified into non-PSCI and PSCI groups based on their test results in the comprehensive neuropsychological battery at 3–6 months after stroke. Between-group comparisons were conducted using the independent sample two-tailed *t*-test or the Mann–Whitney *U* test for continuous variables, depending on whether the data were normally distributed. For categorical variables, we used the *χ*^2^ or Fisher’s exact test, as appropriate. Variables with *P*-values < 0.1 in the univariate analysis were subsequently included in a binary logistic regression model in an attempt to determine variables that can predict PSCI 3–6 months after stroke. In the primary analysis, we used receiver operating characteristic (ROC) curve and area under the curve [AUC; with 95% confidence intervals (CIs)] analyses to compare the discriminatory ability of the MMSE and the MoCA. Also, the ROC curve of the education-adjusted MMSE score was performed by using the regression analysis. Given that both the MMSE and the MoCA showed a good ability for predicting PSCI during the acute phase, we determined optimal predictive values by calculating the maximum value of the Youden index. In the secondary analysis, we used the classification and regression tree (CART) methodology to further validate risk factors that might be associated with cognitive impairment after stroke. Although decision trees and logistic regression models can both be used to identify risk factors, the main advantage of a decision tree is that it allowed us to rank the relative importance of risk factors and create a predictive model. All statistical analyses were performed with IBM SPSS Statistics Version 23 (IBM Corp., Armonk, NY, USA). *P* < 0.05 was considered to indicate statistically significant results.

## Results

### Demographics

**Figure 1:**
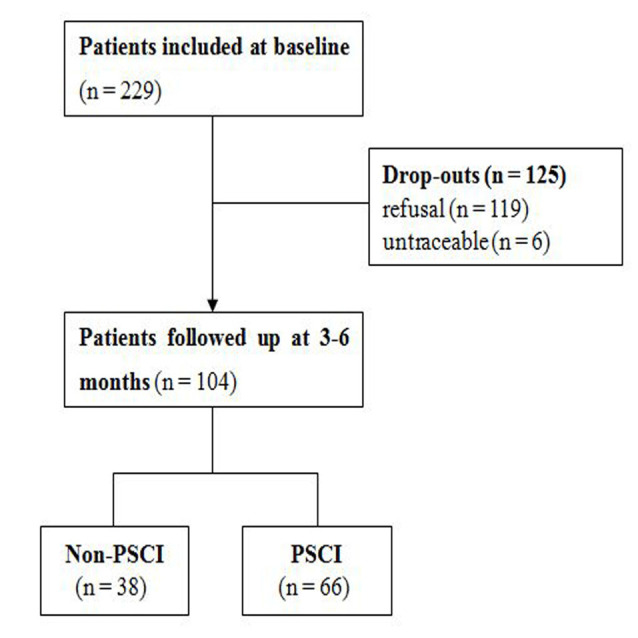
Flowchart depicting the study.

During the inclusion period, a total of 229 acute patients experienced their first-ever ischemic stroke and met the entry criteria for recruitment. Subsequently, 104 of these patients received a comprehensive neuropsychological battery 3–6 months after the onset of stroke and were included in the final analysis (Figure 1). The mean age (±standard deviation, SD) of patients with follow-up was 64.0 ± 9.8 years (range: 36–84 years). The mean age (±SD) of 125 patients lost to follow-up was 60.6 ± 9.6 years (range: 35–83 years). There were no significant differences between patients with or without follow-up in the baseline characteristics, including sex, education, BMI, baseline MMSE scores, and baseline MoCA scores (Supplementary Table S1). At follow-up, the median MMSE score (±interquartile range) had improved from 27.5 ± 3 to 28.0 ± 3.0 (*P* = 0.228), whereas the median MoCA score (± interquartile range) had improved from 22.0 ± 8.0 to 24.0 ± 6.0 (*P* = 0.011). In total, 66 (63.5%) and 38 patients (36.5%) were assigned to the PSCI and non-PSCI groups, respectively.

As shown in Table 1, patients with PSCI had lower educational levels, higher BMIs, lower baseline MMSE scores, and lower baseline MoCA scores (*P* < 0.05). There were no significant differences between the PCSI and non-PCSI groups about lesion location, NIHSS scores, mRS scores, or ApoE genotype. Logistic regression analysis showed that educational level, BMI, and baseline MoCA scores were significant independent variables (*P* < 0.05).

**Table 1:** Baseline characteristics for the two study groups.

	Non-PSCI group (*n* = 38)	PSCI group (*n* = 66)	Univariate analysis *P*-value	Logistic regression *P*-value
Demographics			
Age, years (mean ± SD)	64.3 ± 11.8	63.8 ± 8.6	0.823	
Sex, female (%)	10 (26.3)	24 (36.4)	0.293	
Education, years (median ± IQR)	11 ± 6	6 ± 7	<0.0001*	0.022*
Cardiovascular risk factors				
Hypertension (%)	27 (71.1)	46 (69.7)	0.884	
Diabetes mellitus (%)	10 (26.3)	28 (42.4)	0.1	
Hyperlipidemia (%)	15 (39.5)	33 (50)	0.3	
Atrial fibrillation (%)	3 (7.9)	2 (3.0)	0.352	
Smoking history (%)	19 (50)	34 (51.5)	0.882	
BMI, kg/m^2^ (mean ± SD)	23.5 ± 2.7	25.0 ± 2.7	0.006*	0.038*
Lesion location			0.552	
Left hemisphere (%)	6 (15.8)	11 (16.7)		
Right hemisphere (%)	11 (28.9)	14 (21.2)		
Corpus callosum (%)	0 (0)	2 (3.0)		
Basal ganglia (%)	9 (23.7)	8 (12.1)		
Thalamus (%)	4 (10.5)	8 (12.1)		
Brainstem (%)	5 (13.2)	16 (24.2)		
Cerebellum (%)	3 (7.9)	7 (10.6)		
Stroke severity			
Baseline NIHSS score (median ± IQR)	1 ± 2	1 ± 2	0.768	
Baseline mRS score (median ± IQR)	2 ± 2	2 ± 2	0.103	
Cognitive screening tests				
Baseline MMSE score (median ± IQR)	29 ± 2	26 ± 3	<0.0001*	0.349
Baseline MoCA score (mean ± SD)	25.0 ± 2.7	20.1 ± 4.4	<0.0001*	0.016*
Laboratory tests				
HbA1c, % (median ± IQR)	6.0 ± 0.8	6.3 ± 2	0.098	0.112
Homocysteine, μmol/L (median ± IQR)	13.3 ± 4.6	12.4 ± 3.9	0.357	
APOE ε4-positive (%)	6 (15.8)	11 (16.7)	0.907	

SD, standard deviation; IQR, interquartile range; BMI, body mass index; NIHSS, National Institutes of Health Stroke Scale; mRS, modified Rankin Scale; MMSE, Mini-Mental State Examination; MoCA, Montreal Cognitive Assessment; HbA1c, glycosylated hemoglobin; *P < 0.05.

### Primary Analysis

**Figure 2:**
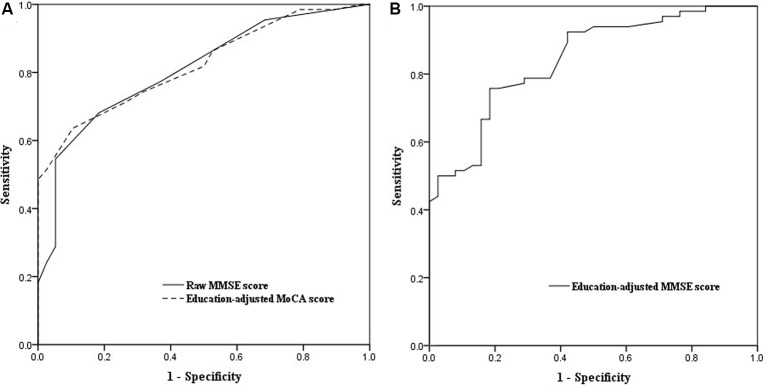
**(A)** Receiver operating characteristic (ROC) curves of raw Mini-Mental State Examination (MMSE) score and education-adjusted Montreal Cognitive Assessment (MoCA) score in the prediction of cognitive impairment at 3–6 months after the onset of stroke. **(B)** ROC curve of education-adjusted MMSE score in the prediction of cognitive impairment at 3–6 months after the onset of stroke.

ROC curve analysis showed that baseline MoCA scores exhibited a higher AUC for predicting PSCI 3–6 months after stroke (AUC, 0.821; 95% CI, 0.743–0.898; Figure 2A) than baseline MMSE scores (AUC, 0.809; 95% CI, 0.725–0.892), although there was no significant difference between the two AUCs (*P* = 0.75). We further performed the ROC curve of education-adjusted MMSE score (AUC, 0.842; 95% CI, 0.767–0.917; Figure 2B), which was similar compared with the ROC curve of raw MMSE score. Due to the lack of statistical equation among Chinese patients, the optimal cut-off score for education-adjusted MMSE cannot be calculated. For raw MMSE data, the optimal cut-off score for the MMSE was 27 (sensitivity, 0.682; specificity, 0.816); and for education-adjusted MoCA data, the optimal cut-off score for the MoCA was 21 (sensitivity, 0.636; specificity, 0.895; Table 2).

**Table 2:** MMSE and MoCA scores during the acute phase for predicting post-stroke cognitive impairment (PSCI) at 3–6 months after the onset of stroke.

Cut-off*	Sensitivity	Specificity	PPV	NPV	Accuracy
MMSE					
25	0.288	0.947	0.905	0.434	0.529
26	0.545	0.947	0.947	0.545	0.692
27^†^	0.682	0.816	0.865	0.596	0.731
28	0.773	0.632	0.785	0.615	0.721
29	0.955	0.316	0.708	0.800	0.721
MoCA					
19	0.485	1.000	1.000	0.528	0.673
20	0.515	0.974	0.971	0.536	0.683
21^†^	0.636	0.895	0.913	0.586	0.731
22	0.712	0.737	0.825	0.596	0.721
23	0.742	0.684	0.803	0.605	0.721

PPV, positive predictive value; NPV, negative predictive value. *Positive test when score ≤ cut-off; ^†^optimal cut-off score calculated using the Youden index.

### Secondary Analysis

The CART methodology was used to create a decision tree incorporating education, BMI, baseline MMSE scores, baseline MoCA scores, and glycosylated hemoglobin levels. Finally, the variables baseline MoCA scores, education, BMI, and baseline MMSE scores remained in the model (Supplementary Figure S1). The decision tree had a sensitivity of 80.3% (53/66), a specificity of 84.2% (32/38), and an AUC of 82.3%. Starting with the “root” node, the 104 included patients were divided into two subsequent “daughter” nodes; the final model featured five terminal nodes. Overall, 91.3% of patients (42/46 cases) with baseline MoCA score ≤21 had PSCI. This analysis identified baseline MoCA scores as the most important predictive factor, followed by educational level, BMI, and baseline MMSE scores. Based on these branch points, patients were stratified into three risk groups for PCSI (high, moderate, and low; Table 3).

**Table 3:** Three risk groups for predicting PSCI, as determined by a decision tree model.

Risk groups	Variables
High (70–100%)	
	Baseline MoCA score ≤21
	Baseline MoCA score >21, education ≤10, BMI >24.815
Moderate (30–70%)	
	Baseline MoCA score >21, education ≤10, BMI ≤24.815
	Baseline MoCA score >21, education >10, baseline MMSE score ≤28
Low (≤30%)	
	Baseline MoCA score >21, education >10, baseline MMSE score >28

*MMSE, Mini-Mental State Examination; MoCA, Montreal Cognitive Assessment; BMI, body mass index; Y, years*.

## Discussion

This is the first study to explore the discriminatory ability of the MMSE and the MoCA to predict cognitive impairment in Chinese patients 3–6 months after minor stroke, when administered during the acute phase and combined with a comprehensive neuropsychological battery. Our analysis revealed two major findings. First, we found that the early application of the MMSE and the MoCA can play a significant role in identifying PSCI. Secondly, we found that baseline MMSE scores, baseline MoCA scores, educational level, and BMI were all risk factors for PSCI.

It is well known that the main limitation of the MMSE is the presence of a ceiling effect due to the lack of accessibility to executive functions. Compared with the MMSE, the MoCA is more suitable for educated individuals, although a floor effect is evident for patients with a lower educational level. Nevertheless, we found that both the MMSE and the MoCA are good screening instruments for PSCI. Patients with an MMSE score ≤27 or a MoCA score ≤21 at baseline were at greater risk of cognitive impairment. The AUCs for the MMSE and the MoCA were similar (0.809 and 0.821, respectively). The optimal cut-off score of the MoCA had higher specificity (0.895) and a lower sensitivity (0.636) than those of the optimal MMSE cut-off score (specificity, 0.816; sensitivity, 0.682).

In our secondary analysis, we created a decision tree that included education, BMI, baseline MMSE scores, and baseline MoCA scores. The generation of this tree led to some improvements in predictive value and provided a ranking for the risk factors associated with PSCI. This analysis also suggested that MoCA plays a significant role in the prediction of PSCI; this is in line with findings from previous studies (Salvadori et al., 2013; Zietemann et al., 2018). Higher education and lower BMI were associated with a reduced risk of cognitive impairment in minor stroke patients. It is well established that a high level of education acts as a protective factor against cognitive deficits in minor stroke patients; this thought to be related to preexisting cognitive processes or due to the development of new compensatory processes (Nunnari et al., 2014). Our analysis showed that a higher BMI was related to an increased risk of PSCI and that the mean age of our patients was 64.0 years. This is consistent with the findings of a previous study that showed that midlife obesity was related to a higher risk of cognitive decline and that underweight people aged >65 years were more prone to suffering from cognitive impairment (Fitzpatrick et al., 2009).

Our study has several limitations that need to be considered. First, bias may have resulted from attrition; 55% of the patients recruited at baseline did not complete the comprehensive neuropsychological battery. Furthermore, some patients with poor prognosis were not available for follow-up analysis. Consequently, the incidence of PSCI may have been underestimated. Furthermore, some patients with a good prognosis may have thought that there was no need to be followed up because their daily life activities had not been influenced by cognitive function; thus, the incidence of PSCI may have alternatively been overestimated. Second, we excluded patients that were unavailable for cognitive screening tests at baseline; this may have led to an underestimation of the incidence of PSCI. Given that the patients included were with low baseline NIHSS scores, the result of our study may only be suitable for minor stroke patients. Future studies should explore the use of other screening tests or predictive indicators that are more feasible. Finally, we did not analyze neuroimaging characteristics in the present study because of a lack of relevant data, including cortical thickness and white matter hyperintensities, as determined by MRI (Dickie et al., 2020). We believe that it would be of great value to investigate whether such MRI characteristics could contribute to the prediction of PSCI in future studies.

In conclusion, because of the high prevalence of cognitive impairment after stroke, it is vital to develop an early screening tool that is effective and easy to apply. Our current findings provide evidence to recommend the MMSE and MoCA as early screening instruments in the acute phase after minor ischemic stroke. We also encourage the use of a comprehensive neuropsychological battery for high-risk patients (those with an MMSE score ≤27 or a MoCA score ≤21) to predict PSCI 3–6 months after the onset of stroke.

## Data Availability Statement

All datasets generated for this study are included in the article/Supplementary Material.

## Ethics Statement

The studies involving human participants were reviewed and approved by the Human Ethics Review Committee of the Second Affiliated Hospital, Zhejiang University School of Medicine. The patients/participants provided their written informed consent to participate in this study.

## Author Contributions

YZ designed the study, collected and analyzed the data, and drafted the manuscript. SZ, ZF, ZLi, FH, CL, WT, YY, and ZLiu collected and analyzed the data. YC and BZ designed the study and revised the manuscript. All authors contributed to the article and approved the submitted version.

## Conflict of Interest

The authors declare that the research was conducted in the absence of any commercial or financial relationships that could be construed as a potential conflict of interest.
