# Supplementary material for: Evaluation of the Mini-Mental State Examination and the Montreal Cognitive Assessment for Predicting Post-stroke Cognitive Impairment During the Acute Phase in Chinese Minor Stroke Patients
Source: Front Aging Neurosci. 2020 Aug 6;12:236. doi: 10.3389/fnagi.2020.00236 (PMC7424073; doi:10.3389/fnagi.2020.00236)
Supplement: Supplementary file 1 [file Table_1.pdf]

Supplementary material

Table S1 Baseline Characteristics of patients with and without follow-up.

	Patients with follow-up (n=104)	Patients without follow-up (n=125)	*P* value
Age, y (mean ± SD)	64.0 ± 9.8	60.6 ± 9.6	0.009*
Sex, female (%)	34 (32.7)	45 (36.0)	0.600
Education, y (median ± IQR)	8.5 ± 7.0	7.0 ± 8.0	0.465
BMI, kg/m^2 (mean ± SD)	24.4 ± 2.8	24.4 ± 3.1	0.924
Baseline NIHSS score (median ± IQR)	1.0 ± 2.0	1.0 ± 2.0	0.038*
Baseline MMSE score (median ± IQR)	27.5 ± 3.0	27.0 ± 3.0	0.097
Baseline MoCA score (mean ± SD)	22.0 ± 8.0	22.0 ± 6.0	0.385

SD, standard deviation; IQR, inter-quartile range; BMI, body mass index; NIHSS, National Institutes of Health Stroke Scale; MMSE, Mini-Mental State Examination; MoCA, Montreal Cognitive Assessment; *$P < 0.05$.
