# Supplementary material for: Evaluation of the Mini-Mental State Examination and the Montreal Cognitive Assessment for Predicting Post-stroke Cognitive Impairment During the Acute Phase in Chinese Minor Stroke Patients
Source: Front Aging Neurosci. 2020 Aug 6;12:236. doi: 10.3389/fnagi.2020.00236 (PMC7424073; doi:10.3389/fnagi.2020.00236)
Supplement: Supplementary file 2 [file Image_1.pdf]

Supplementary material

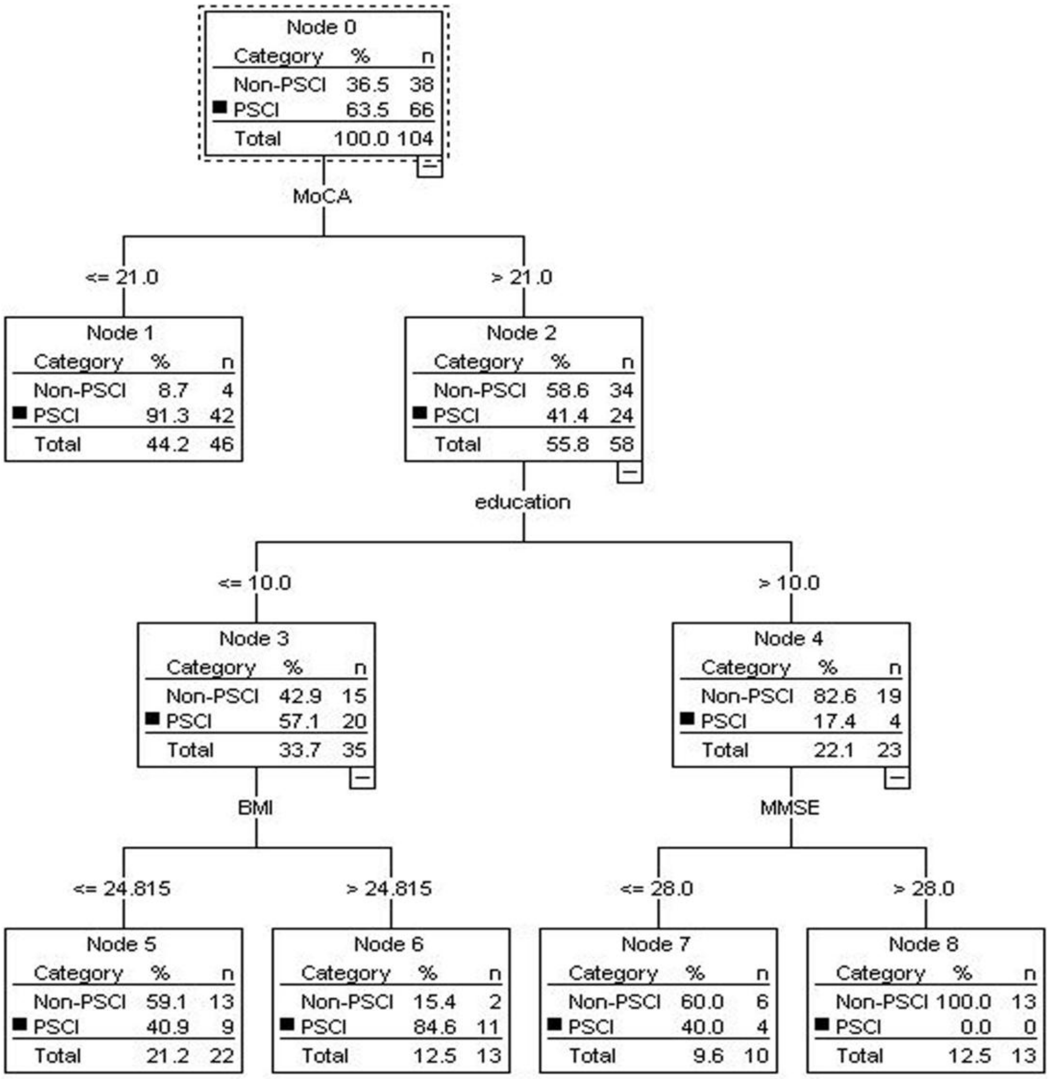

Figure S1. A decision tree model for the prediction of cognitive impairment at 3 to 6 months after the onset of stroke that includes baseline MoCA scores, educational level, body mass index (BMI), and baseline MMSE scores.
